# Phosphorylation modifies the molecular stability of β-amyloid deposits

**DOI:** 10.1038/ncomms11359

**Published:** 2016-04-13

**Authors:** Nasrollah Rezaei-Ghaleh, Mehriar Amininasab, Sathish Kumar, Jochen Walter, Markus Zweckstetter

**Affiliations:** 1 German Center for Neurodegenerative Diseases (DZNE), 37075 Göttingen, Germany; 2 Department of Cell and Molecular Biology, School of Biology, College of Science, University of Tehran, 1417466191 Tehran, Iran; 3 Department of Neurology, University of Bonn, 53127 Bonn, Germany; 4 Department for NMR-based Structural Biology, Max Planck Institute for Biophysical Chemistry, 37077 Göttingen, Germany; 5 Department of Neurology, University Medical Center Göttingen, University of Göttingen, 37073 Göttingen, Germany

## Abstract

Protein aggregation plays a crucial role in neurodegenerative diseases. A key feature of protein aggregates is their ubiquitous modification by phosphorylation. Little is known, however, about the molecular consequences of phosphorylation of protein aggregates. Here we show that phosphorylation of β-amyloid at serine 8 increases the stability of its pathogenic aggregates against high-pressure and SDS-induced dissociation. We further demonstrate that phosphorylation results in an elevated number of hydrogen bonds at the N terminus of β-amyloid, the region that is critically regulated by a variety of post-translational modifications. Because of the increased lifetime of phosphorylated β-amyloid aggregates, phosphorylation can promote the spreading of β-amyloid in Alzheimer pathogenesis. Our study suggests that regulation of the molecular stability of protein aggregates by post-translational modifications is a crucial factor for disease progression in the brain.

## Abstract (web summary)

Protein aggregation plays a crucial role in several neurodegenerative diseases. Here the authors demonstrate that phosphorylation of β-amyloid aggregates—the pathological hallmark of Alzheimer's disease—can change the molecular properties of aggregates, suggesting how phosphorylation contributes to disease progression.

## Graphical abstract

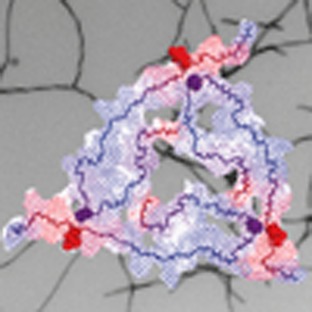

Protein aggregation plays a crucial role in several neurodegenerative diseases such as Alzheimer (AD) and Parkinson disease^1^. A remarkable feature of neurotoxic protein aggregation is the ubiquitous presence of post-translational modifications of aggregated proteins^2^,^3^. A particular abundant modification of amyloid deposits is phosphorylation^4^. Phosphorylation influences protein–protein and protein–lipid interactions, protein turnover and subcellular localization of neurodegeneration-related proteins^4^,^5^,^6^,^7^,^8^. In addition, phosphorylation plays a significant role in regulating the clearance mechanisms of proteinaceous deposits^4^,^7^,^9^,^10^. Despite its relevance in neurodegenerative diseases, the effect of phosphorylation on the molecular properties of amyloid deposits is largely unknown.

Protein amyloid fibrils exhibit remarkable stability against physical perturbations suggesting that they may represent the lowest free-energy state in the conformational energy landscape of proteins^11^,^12^. Amyloid fibrils also possess intriguing mechanical properties with an elastic Young's modulus comparable to that of the most rigid proteinaceous materials in nature^13^. The nanomechanical features of amyloid fibrils influence the kinetics of fibril growth by modulating their inherent fragmentation tendency^14^. Fragmentation of protein fibrils is therefore thought to be crucial for propagation and spreading of disease pathology^15^. Less ordered but more toxic aggregate species such as protofibrils and early oligomeric assemblies often have lower physical and mechanical stability than fully formed fibrils^13^,^16^,^17^, further supporting a strong link between the stability of protein aggregates and their pathological function^14^.

Early-onset familial forms of AD are generally caused by mutations, which increase amyloid β (Aβ) production, elevate Aβ42/Aβ40 ratio or enhance Aβ propensity for formation of toxic aggregates^18^. In contrast, causes of the much more common late-onset sporadic AD are poorly understood. It has been suggested that post-translational modifications such as phosphorylation, nitration and pyroglutamination may play an important role in the initiation and progression of sporadic AD^4^,^19^,^20^,^21^. Indeed, extracellular Aβ can undergo phosphorylation by a cell surface-localized or secreted form of protein kinase A and Aβ phosphorylated at serine 8 is detectable in human AD brain^22^,^23^. Phosphorylation of Aβ at serine 8 markedly accelerates aggregation of Aβ *in vitro* and *in vivo* and increases Aβ-mediated toxicity in *Drosophila* models of AD^22^. In addition, it attenuates proteolytic degradation of Aβ by certain proteases^24^.

Here we investigate how phosphorylation at serine 8 alters the kinetic and thermodynamic stability of Aβ aggregates. Using high-pressure nuclear magnetic resonance (NMR) and molecular dynamics (MD) simulation, we demonstrate that phosphorylation modifies the stability of amyloid aggregates of Aβ and propose a structural basis for the higher stability of phosphorylated Aβ aggregates. Our study supports an important role of phosphorylation and other post-translational modifications of amyloid deposits in neurodegenerative diseases.

## Results

### Aβ aggregates are sensitive to pressure

To investigate the effect of phosphorylation on the stability of Aβ aggregates, we employed high pressure as a tool to induce protein disaggregation. In general, the susceptibility of protein aggregates to high pressures is largely governed by their degree of compaction^25^,^26^,^27^. Mature amyloid fibrils are largely resistant to pressure, because tight packing of their backbone and side-chain groups minimizes void volumes inside the fibrils. In contrast, aggregates, which appear early during amyloid aggregation, are often rich of internal water-excluded cavities^28^, rendering them sensitive to pressure-induced dissociation^11^,^16^. In addition, ionic interactions often experience significant volume decrease on disruption and are therefore sensitive to high pressure^29^. To assist pressure-induced dissociation of Aβ aggregates, we performed the experiments at 0 °C.

**Figure 1:**
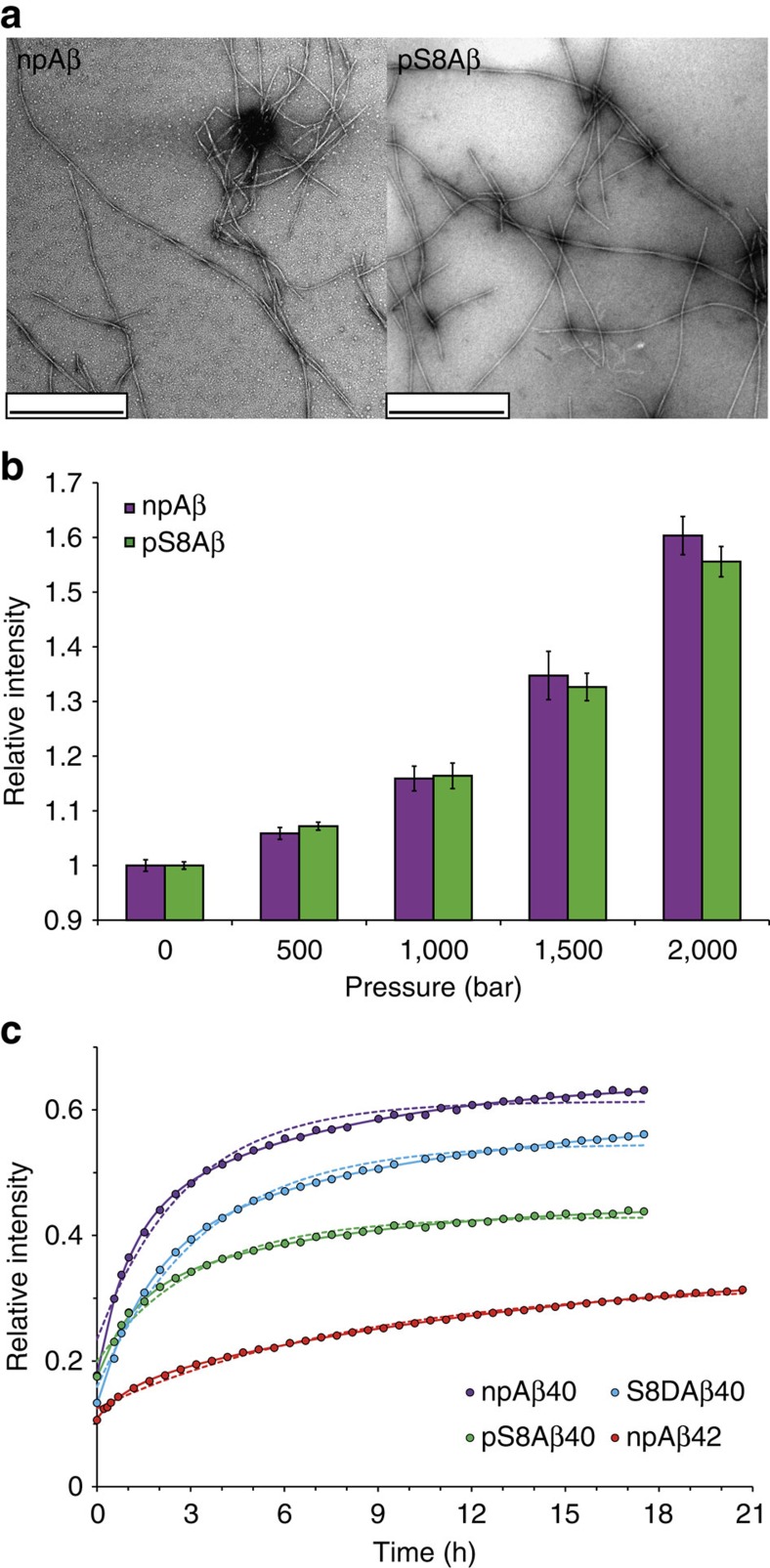
Aβ aggregates have distinct pressure stability. (**a**) Electron micrographs of aggregated Aβ40 in its non-phosphorylated (npAβ) and S8-phosphorylated (pS8Aβ) state reveal amyloid fibrils of similar morphology. Scale bar, 500 nm. (**b**) Pressure dependence of Aβ monomer release from npAβ (magenta) and pS8Aβ (green) aggregates. Error bars represent the s.d. between three consecutive measurements each taking 10 min. (**c**) Time dependence of Aβ monomer release, as followed by NMR signal intensities, on incubation of aggregated Aβ at a hydrostatic pressure of 2,000 bar. Data fit to mono- and bi-exponential functions are presented in dotted and solid lines, respectively. Phosphorylation at S8 attenuates the monomer release from Aβ40 aggregates. In addition, Aβ42 aggregates (red) are more stable than Aβ40 aggregates.

**Figure 2:**
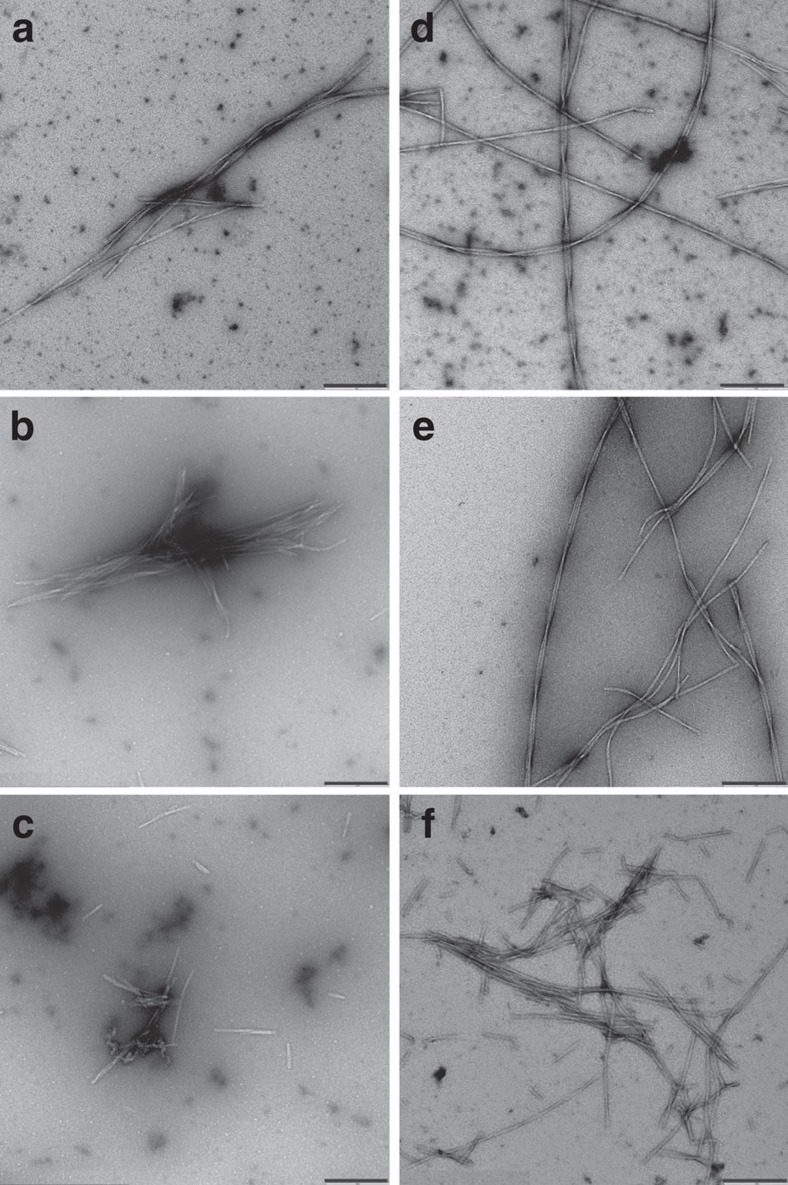
Effect of high pressure on Aβ fibrils. Electron micrographs of npAβ (**a**–**c**) and pS8Aβ (**d**–**f**) aggregates, before (**a**,**d**) and after (**b**–**c**,**e**–**f**) application of high hydrostatic pressure (2,000 bar, 18 h). Many fibrils were unaffected by the high pressure (**b**,**e**), but some broken fibrils were also observed (**c**,**f**). Scale bar, 200 nm.

Wild-type Aβ (npAβ) and Aβ phosphorylated at serine 8 (pS8Aβ) were incubated in aggregation-prone conditions for 4 days, which led to the formation of mature amyloid fibrils (Fig. 1a). Subsequently the aggregates were exposed to increasing pressures up to 2,000 bar and the release of Aβ monomers was monitored by one-dimensional proton NMR spectroscopy. Figure 1b demonstrates that Aβ monomers were released from the preformed aggregates of both npAβ and pS8Aβ in dependence of the applied pressure. Consistent with a pressure-induced perturbation of the aggregates, a slight reduction in the ThT fluorescence signal intensities was observed after pressure application (Supplementary Fig. 1). The drop in ThT intensity was lower than the level of concomitant Aβ monomer release suggesting that the released Aβ monomers originated partly from non-ThT reactive aggregates. Application of pressure up to 2,000 bar did not have a large effect on the morphology Aβ fibrils, although a number of broken fibrils were detected after pressure application (Fig. 2).

### Phosphorylation modifies the stability of Aβ aggregates

For both npAβ and pS8Aβ, incubation in aggregation-prone conditions for 96 h decreased the NMR signal intensity to ∼18% of the initial value, indicating conversion of >80% of Aβ molecules to NMR-invisible aggregates. We then followed the kinetics of Aβ monomer release at 2,000 bar: after ∼18 h of incubation at 2,000 bar, the NMR intensity increased from 18 to ∼63% for npAβ and to ∼44% for pS8Aβ (Fig. 1c). To quantify the release of Aβ monomers from the aggregates, we fitted the gradual rise in NMR signal intensities to a monoexponential function. From the analysis an apparent rate *R* of 0.79±0.04 × 10^−4^ s^−1^ and limiting intensity value of 0.62 were obtained for npAβ40. In case of pS8Aβ40, values of 0.73±0.04 × 10^−4^ s^−1^ and 0.43 were calculated. Notably, the quality of fit was significantly improved when a bi-exponential function was used (Fig. 1c; Supplementary Table 1).

**Figure 3:**
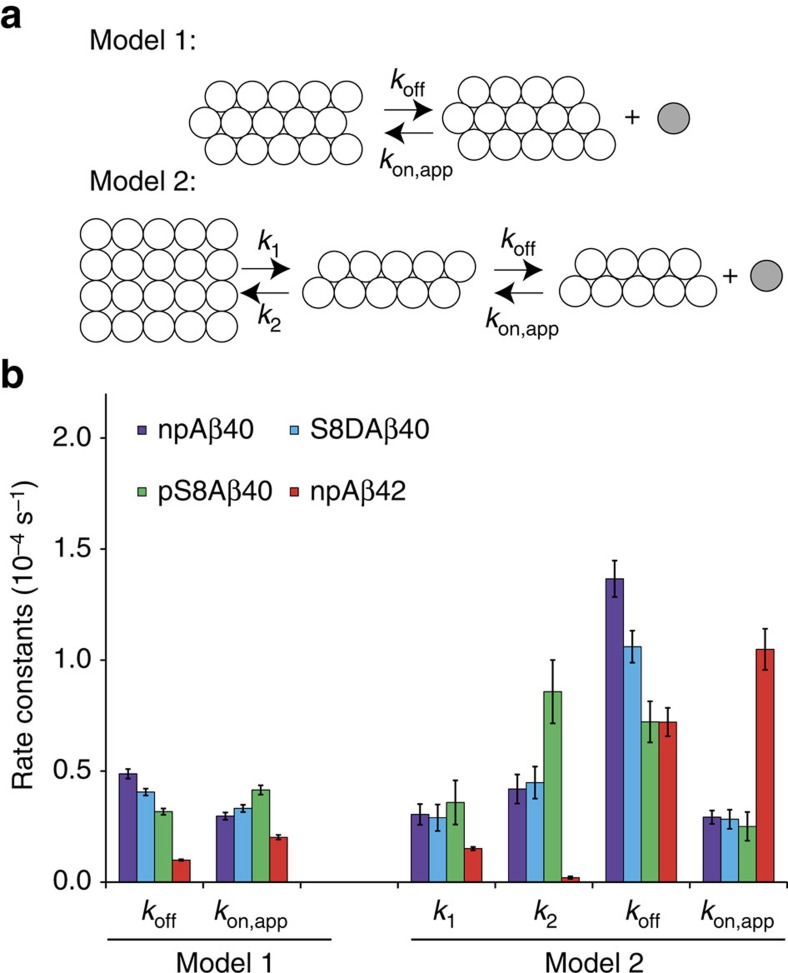
Phosphorylation modulates the kinetic and thermodynamic stability of Aβ aggregates. (**a**) Schematic representation of two kinetic models for aggregate dissociation. Model 1 consists of a single step of reversible Aβ monomer (grey) dissociation from aggregates. Model 2 involves a reversible step in which Aβ aggregates are converted to an intermediate state under rate constants *k*_1_ and *k*_2_, followed by a second reversible step of monomer release from the intermediate state. (**b**) Rate constants obtained from the analysis of monomer release kinetics (Fig. 1c) according to the two kinetic models shown in **a**. Error bars represent s.e.s of fitting parameters.

To interpret the fitting parameters in terms of physical rates, we first assumed the simplest model involving only a single step of reversible Aβ monomer dissociation from aggregates (Fig. 3a). Assuming that the association reaction obeys (pseudo) first-order kinetics in terms of monomer concentration, the rates of monomer dissociation (*k*_off_) of 0.49±0.02 × 10^−4^ s^−1^ for npAβ and 0.32±0.01 × 10^−4^ s^−1^ for pS8Aβ were obtained. The corresponding association rates (*k*_on,app_) were 0.30±0.02 × 10^−4^ s^−1^ (npAβ) and 0.42±0.05 × 10^−4^ s^−1^ (pS8Aβ). The obtained *k*_off_ rate of 0.49±0.02 × 10^−4^ s^−1^ for npAβ is comparable to the off-rate value of 1.2 × 10^−4^ s^−1^ previously reported for the protofibrils of wild-type Aβ40 (25 °C, ambient pressure) on the basis of fluorescence measurements^30^. In case of amyloid fibrils formed by an SH3 domain (28 °C, ambient pressure), a dissociation rate of 1.4 × 10^−4^ s^−1^ had been estimated^31^. Compared with the extrapolated *k*_off_ value of ∼0.06 × 10^−4^ s^−1^ at 0 °C in ref. 30, the use of high pressure in our study has resulted in an about eightfold increase in the monomer dissociation rate of npAβ.

In the next step, we considered a more complex model involving two steps: first, a reversible step in which Aβ aggregates are converted to an intermediate state under rate constants *k*_1_ and *k*_2_, and second, the reversible monomer dissociation from these intermediate aggregates governed by the rate constants *k*_off_ and *k*_on,app_ as described above (Fig. 3a). This model implies bi-exponential behaviour of Aβ monomer level in response to increased pressure, which was shown above to better fit to the experimental data than the monoexponential model (Fig. 1c; Supplementary Tables 1 and 2). Serine 8 phosphorylation did not have a significant effect on the forward kinetics of the first step, but diminished *k*_off_ rate in the second step (Fig. 3b; Supplementary Table 2). The lower level of pressure-induced monomer release from pS8Aβ than from npAβ aggregates provides substantial support for an enhanced stability of Aβ aggregates in the S8-phosphorylated state. In addition, the decrease in the monomer dissociation rate (*k*_off_) for both models demonstrates the increased kinetic stability of phosphorylated Aβ aggregates.

Aspartate variants are widely used to model defined phosphorylation states of neurotoxic proteins *in vivo*. Indeed, replacement of serine 8 by aspartate in Aβ promotes its aggregation similar to pS8Aβ (Supplementary Fig. 2)^22^. We therefore asked whether the S8D substitution could also mimic the effect of S8-phosphorylation on the stability of Aβ aggregates. To this end, we exposed S8DAβ aggregates to high pressure. Figure 1c shows that the level of pressure-induced monomer release from S8DAβ aggregates was in between that of npAβ and pS8Aβ (Supplementary Table 1). Comparison of the kinetic parameters of npAβ, S8DAβ and pS8Aβ monomer release demonstrated that the off-rate *k*_off_ was similarly reduced in S8DAβ albeit at a smaller level than pS8Aβ (Fig. 3b; Supplementary Table 2). In contrast, no significant changes in the apparent on-rates *k*_on,app_ were observed (Fig. 3b). This suggests differences between phosphomimetic variants and naturally occurring phosphorylated proteins even when, as it is the case for the pS8Aβ and S8DAβ, the general aggregation behaviour of phosphorylated and phosphomimetic protein is similar.

### Volume change on Aβ monomer release from aggregates

**Figure 4:**
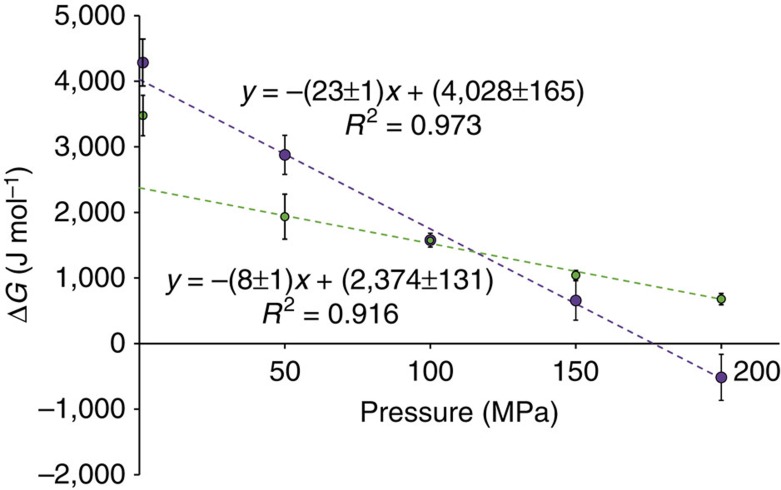
Volume change on Aβ monomer release from aggregates. The pressure dependence of free-energy change on monomer dissociation from npAβ40 (magenta) and pS8Aβ40 (green) aggregates is shown. Slopes of fitted lines represent volume changes on Aβ monomer release from aggregates, which is approximately three times smaller in pS8Aβ40. Error bars indicate 1 s.d. of 2–5 repeated measurements.

To gain insight on the impact of S8 phosphorylation on volume changes during Aβ monomer release, we monitored how the equilibrium between aggregated and monomeric Aβ is affected by pressure. After 2 days of pressure application to aggregated Aβ samples (at 500, 1,000, 1,500 and 2,000 bar, 0 °C), the Aβ monomer levels were quantified by NMR and the equilibrium constants between Aβ aggregates and monomers derived (equation (3)). As shown in Fig. 4, the pressure dependence of free-energy change on monomer release from npAβ40 aggregates (Δ*G*_A→M_) resulted in an apparent volume change of 23±1 μl mol^−1^. Since the free-energy change at ambient pressure was quite close to the intercept of the fitted line, the volume change on monomer release seemed to be effectively constant over the studied pressure range. On the other hand, the volume change on monomer release from pS8Aβ40 aggregates was only 8±1 μl mol^−1^, three times lower than that of npAβ40. Notably, the free-energy change at ambient pressure was significantly above the fitted line (green data point at ambient pressure in Fig. 4), indicating that the volume change on monomer release was decreased by pressure, that is, the compressibility of pS8Aβ40 aggregates in water was higher than its monomers.

### Mechanistic basis of altered stability of amyloid fibrils

Despite the drastically higher rate and amount of amyloid aggregation of pS8Aβ, the morphology of fibrils differed only subtly from that of npAβ (Fig. 1a). In the negatively stained transmission electron micrographs, the fibrils formed by the two peptides were similarly twisted and their apparent diameter (maximum width) was 13.7±3.6 nm for npAβ and 11.5±1.9 nm for pS8Aβ. The size and morphology of Aβ40 fibrils resembled those generated in a previous study under quiescent condition and lower temperature of 24 °C (ref. 32). The size distribution of npAβ fibrils exhibited a weakly bimodal behaviour, with the major and minor modes centered at ∼12 and 24 nm, respectively, (Supplementary Fig. 3). Fibrils of pS8Aβ were more strongly centered at the 12-nm mode, suggesting that phosphorylation of serine 8 affected the higher order assembly of Aβ40 fibrils.

To gain insight into the molecular basis of the increased structural stability of Aβ aggregates, we performed MD simulations of Aβ40 fibrils in different phosphorylation states. As starting conformation we selected the threefold symmetric structure of Aβ40 fibrils prepared after seeding by *in vivo* plaques^33^. This structure was chosen because it is currently the only available experimental model of Aβ fibrils, which contains detailed coordination information about the structure of the N-terminal residues of Aβ. The high level of similarity in fibrillar morphology of npAβ and pS8Aβ (Fig. 1a; Supplementary Fig. 3) supports the use of same structural model for both species.

**Figure 5:**
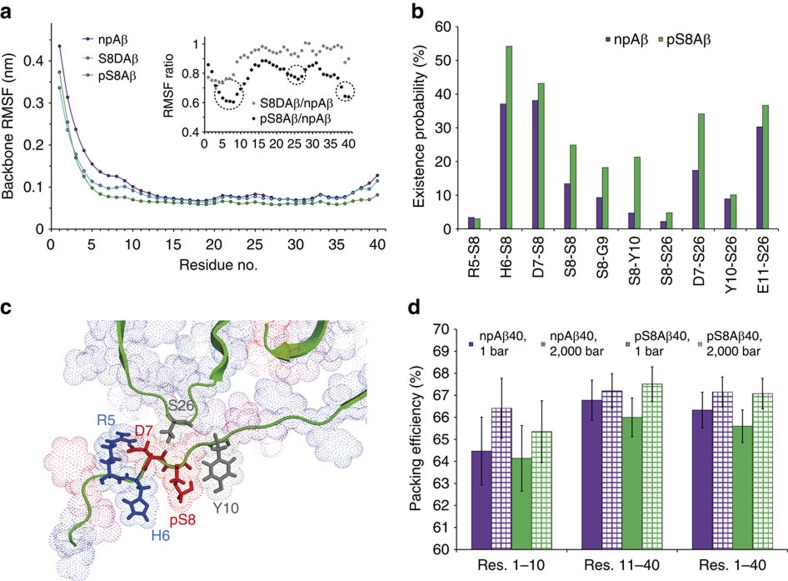
Structural basis for the increased rigidity of Aβ aggregates phosphorylated at serine 8. (**a**) Residue-specific r.m.s. of fluctuations (RMSF) in backbone atom positions averaged over the last 25 ns of MD trajectories of npAβ (magenta), S8DAβ (blue) and pS8Aβ (green) amyloid fibrils. The inset shows the respective ratios with strongly affected regions circled. (**b**) Probability of hydrogen bond formation between the N-terminal residues of Aβ, as well as interstrand hydrogen bond formation with residues S26-N27. Phosphorylation at serine 8 increases the frequency of hydrogen bond formation. (**c**) Favourable electrostatic interactions in the N-terminal region of Aβ40 fibrils phosphorylated at serine 8. (**d**) Packing efficiency of npAβ40 and pS8Aβ40 fibrils at low (1 bar) and high (2,000 bar) pressure, reported separately for residues 1–10 in the N-terminal region, residues 11–40 and the whole Aβ molecule. Compared with npAβ40, the pS8Aβ40 fibrils experience larger pressure-induced increase in packing efficiency, particularly in residues 11–40. Error bars represent one s.d. over the last 25 ns of MD simulation trajectories.

Figure 5a shows how the mobility of the backbone atoms of Aβ40 amyloid fibrils is influenced by the phosphorylation state of serine 8. The most flexible part of the wild-type Aβ40 sequence in the MD simulation is the N-terminal region: the backbone atoms of D1-Y10 experienced high root-mean-square fluctuations (r.m.s.f.) when compared with other parts of Aβ40. When serine 8 was phosphorylated, an overall decrease in the r.m.s.f. of the backbone atoms was observed, but the strongest reduction was found for residues F4-Y10. In addition, V24-N27 and G38-V40 showed a slight drop in r.m.s.f. (Fig. 5a). Mapping of r.m.s.f. values onto a three-dimensional structure of Aβ40 fibrils^33^ showed that the two patches of residues, F4-Y10 and V24-N27 that partially lose their mobility on S8-phosphorylation, are in spatial proximity to the phosphorylation site (Supplementary Fig. 4). Comparison of the average conformation of the last 25 ns of the MD trajectory further highlights the distinct mobility of Aβ40 fibrils when phosphorylated at serine 8 (Supplementary Fig. 5). In case of the phosphomimetic mutation, the reduction in backbone mobility was less pronounced and was restricted more to the N-terminal region (Fig. 5a; Supplementary Fig. 5).

Phosphoserines have a higher capacity for hydrogen bond formation than serine residues, because the phosphate group can act both as donor and acceptor in hydrogen bonds. To see whether the higher rigidity of the N-terminal region in S8-phosphorylated Aβ40 fibrils is caused by the stronger hydrogen bond capacity of phosphoserine, we calculated the existence probability of several hydrogen bonds involving the phosphorylated residue. While the total number of hydrogen bonds in npAβ and pS8Aβ fibrils was highly similar (∼23 for Aβ molecule), phosphorylation of serine 8 enhanced hydrogen bond formation between S8 and the nearby residues R5-Y10 within the same or adjacent Aβ molecules (Fig. 5b). In addition, a long-range hydrogen bond between S8 and S26 was slightly more populated when serine 8 was phosphorylated. Notably, some hydrogen bonds not involving the phosphoserine were also more frequently populated, for example, between D7 and S26, and to a lesser extent between Y10 and N27.

To investigate how S8 phosphorylation influences backbone conformation in the proximity of the phosphorylation site, we compared the intramolecular distance between CA atoms of residues E3-Y10 in npAβ and pS8Aβ fibrils. The average E3CA-Y10CA distance increased from 1.94±0.02 nm in npAβ fibrils to 2.06±0.01 nm in pS8Aβ fibrils, an increase of ∼6% which is probably caused by additional steric demand in the phosphorylated fibrils (change to a fully extended conformation would increase this distance by 30%). In addition, the average structures of the Aβ fibrils over the MD trajectory offer a potential basis for the local conformation (Fig. 5c): a favourable electrostatic interaction between H6 and the phosphorylated S8 leads to the rearrangement of residues R5-Y10. Due to this rearrangement, the electrostatic attraction between the side chains of R5 and D7 is stabilized and the side chains of pS8 and Y10 form a hydrogen bond. In addition, hydrogen bond formation between the backbone atoms of D7 and the side-chain OH of S26 is favoured, contributing to the partial rigidification of S26 and its nearby residues.

### Phosphorylation increases compressibility of Aβ fibrils

To address how S8 phosphorylation influences the volume of npAβ40 and pS8Aβ40 fibrillar state in dependence of pressure, we performed MD simulations at the elevated pressure of 2,000 bar. The packing efficiency of Aβ fibrils was then calculated as the ratio of the van der Waals to Voronoi volumes and compared between low and high pressures. For npAβ40 fibrils, the average packing efficiency increased from 66.3±0.8% at 1 bar to 67.2±0.7% at 2,000 bar, a small rise of ∼1.2% (Fig. 5d). On the other hand, the packing efficiency of pS8Aβ40 fibrils increased from 65.6±0.7% at 1 bar to 67.1±0.7% at 2,000 bar, an increase of 2.3%, which was twice the corresponding value for npAβ40 fibrils. Unlike npAβ40 fibrils, in which the increase in packing efficiency was more prominent in the N-terminal region, the packing efficiency of pS8Aβ40 was enhanced nearly uniformly over the Aβ molecule (Fig. 5d).

As an added mechanism, the ionic interaction between the side chains of H6 and pS8 in phosphorylated Aβ fibrils may contribute to the higher compressibility of pS8Aβ40 fibrils, since breakage of intramolecular electrostatic interactions and resultant electrostriction of solvent molecules by separated charges could decrease the volume of the system without monomer release^29^. We therefore investigated how high pressure altered the distance between neighbouring charges around the phosphorylation site. In line with the increase in packing efficiency at high pressure, the distance between R5 and D7 side chains decreased in both npAβ40 and pS8Aβ40 fibrils. On the other hand, while the distance between H6 and (p)S8 side chains remained intact in npAβ40, it was increased by approximately 8–9% in pS8Aβ40. The relative charge separation induced in pS8Aβ40 fibrils therefore enables the solvent electrostriction effect to further reduce the system volume at high pressure.

### Stability of Aβ aggregates against SDS-induced dissociation

**Figure 6:**
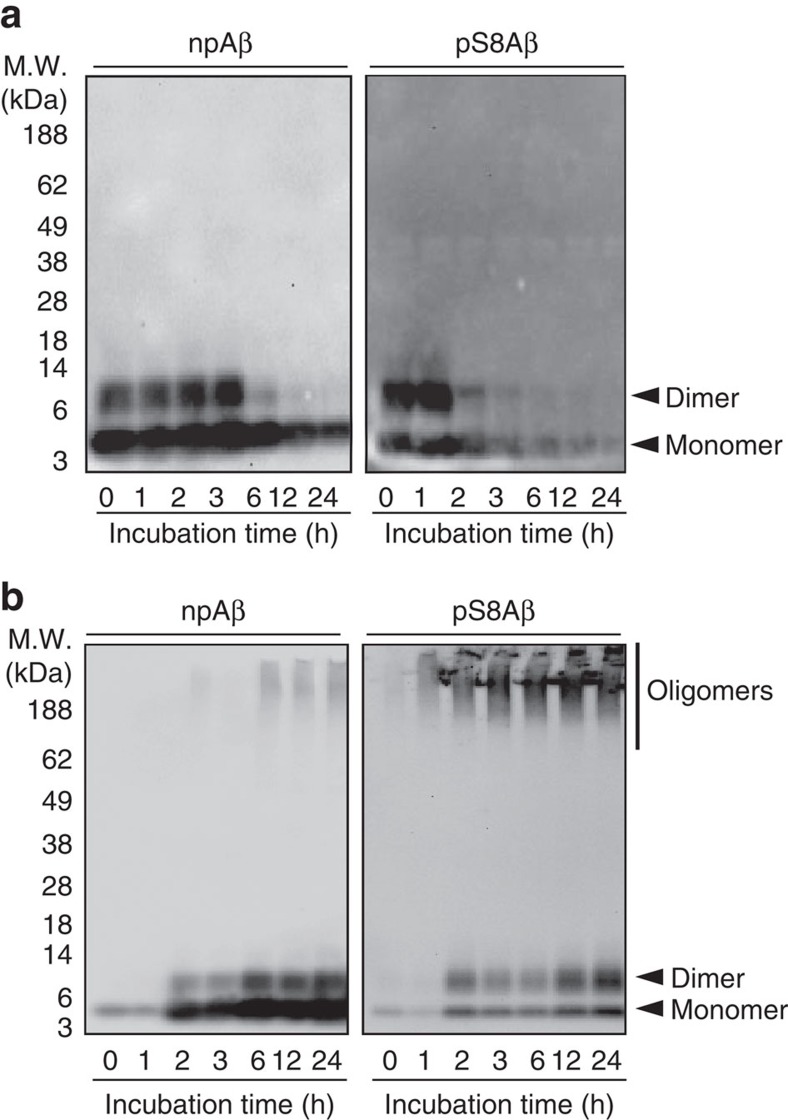
Phosphorylation of Aβ at serine 8 enhances the stability of Aβ oligomers. (**a**) SDS–PAGE of the soluble fraction of aggregated npAβ and pS8Aβ. All soluble oligomers were dissociated under denaturing SDS–PAGE condition and only monomeric and dimeric Aβ was detected. The disappearance of monomeric/dimeric bands occurred more quickly for pS8Aβ than for npAβ, consistent with the higher rate of insoluble Aβ aggregation in case of pS8Aβ. (**b**) SDS–PAGE of the insoluble fraction of aggregated npAβ and pS8Aβ samples demonstrates that high-molecular-weight oligomers of pS8Aβ are more resistant against SDS-induced dissociation.

To further investigate the stability of phosphorylated Aβ assemblies, soluble and insoluble assemblies of pS8Aβ and npAβ were separated by ultracentrifugation after different time points of aggregation and subjected to denaturing SDS–PAGE followed by western blotting. In the soluble fractions, only monomeric and dimeric Aβ40 was observed indicating that soluble oligomers of both npAβ and pS8Aβ were dissociated under denaturing SDS–PAGE conditions (Fig. 6a). In contrast, the insoluble aggregates of pS8Aβ and npAβ showed different stability against SDS-induced dissociation (Fig. 6b; Supplementary Fig. 6). Only very little SDS-resistant oligomeric forms were detected for npAβ, even in samples taken after 12 and 24 h of incubation when robust amounts of ThT-reactive aggregates were present (Supplementary Fig. 7). In contrast, the insoluble assemblies of pS8Aβ were more stable in SDS–PAGE. High-molecular-weight oligomeric bands were observed and the intensity of the monomeric and dimeric bands was decreased in S8-phosphorylated Aβ40. Whether these SDS-stable assemblies of pS8Aβ represent the intermediate oligomeric species populated over the course of aggregation remains, however, to be elucidated.

### Aβ42 aggregates are more stable than Aβ40 aggregates

Between the two abundant variants of Aβ found in human brains, Aβ42 has a stronger aggregation propensity than Aβ40 *in vitro* and *in vivo*, and is more toxic in a variety of animal models of AD^34^. To examine whether the distinct aggregation and toxicity behaviour of Aβ42 is accompanied by differences in the stability of Aβ42 aggregates, we performed high-pressure NMR measurements for Aβ42 aggregates. After 21 h of incubation at 2,000 bar, the NMR signal intensity reached ∼31% of the initial (monomeric) signal intensity, which is by a factor of two smaller than the value of 63% observed for Aβ40 (Fig. 1c; Supplementary Table 1). Consistent with the kinetic data for Aβ40 fibrils, the kinetics of monomer release from Aβ42 aggregates was more suitably described by the two-step model. Analysis of the calculated kinetic parameters revealed that the first step, which involves the reversible conversion of the preformed Aβ aggregates to an intermediate aggregate state was slowed down in Aβ42 when compared with Aβ40. In addition, the off-rate of monomer release from the intermediate Aβ aggregates was reduced, while the rate of monomer association to the intermediate aggregates was increased (Fig. 3b). Taken together the data reveal that Aβ42 aggregates are thermodynamically and kinetically more stable than Aβ40 aggregates.

## Discussion

Phosphorylation of aggregated proteins is the pathological hallmark of a wide variety of neurodegenerative diseases^4^. It affects conformational stability of globular proteins and influences the self- and hetero-association of both globular and intrinsically disordered proteins^4^,^35^. Phosphorylation of Aβ at S26 blocks amyloid fibrillation^36^. In contrast, phosphorylation of Aβ at serine 8 drastically increases the rate and amount of Aβ oligomerization and amyloid fibril formation^22^. Here we demonstrated that Aβ aggregates phosphorylated at serine 8 are more resistant against high-pressure and SDS-induced dissociation than non-phosphorylated aggregates (Figs 1 and 6). A quantitative analysis of pressure-induced monomer release showed that Aβ40 aggregates phosphorylated at serine 8 have higher thermodynamic (*k*_on,app_/*k*_off_) and kinetic (1/*k*_off_) stability than their non-phosphorylated counterparts (Fig. 3). The combination of faster aggregation and higher concentration of oligomeric aggregates results in a higher toxicity of Aβ phosphorylated at serine 8 (ref. 22).

Several lines of evidence attribute an important role for aggregation of Aβ to its N-terminal region^37^,^38^,^39^,^40^. The results from our MD simulation suggested that the high capacity of phosphate groups to act both as the donor and acceptor of hydrogen bonds can lead to the formation of a rich network of hydrogen bonds in the N-terminal region and significantly diminishes backbone dynamics in phosphorylated Aβ aggregates (Fig. 5). To accommodate the bulky phosphate group and maximize the number of stabilizing hydrogen bonds and electrostatic interactions around the phosphoserine, a more extended conformation is induced in the vicinity of the phosphorylation site. We propose that this more extended conformation of the N-terminal region of Aβ may form the structural basis for the enhanced stability of phosphorylated Aβ oligomers. This is in agreement with a recent report showing that the disruption of local extended conformation through S8P mutagenesis leads to selective destabilization of Aβ oligomers^37^.

Pressure-induced dissociation of protein aggregates is governed by volume change on monomer release. Our experimental data demonstrated a volume change of ∼23 μl mol^−1^ for npAβ40, which corresponds roughly to ∼40 Å^3^ per each Aβ molecule (Fig. 4). The corresponding value for pS8Aβ40 was only 8 μl mol^−1^. MD simulations suggested that the lower volume change on pS8Aβ40 monomer release was caused by the higher compressibility of pS8Aβ40 fibrils, which might be attributed to its lower degree of compaction. Another contribution to the higher compressibility of pS8Aβ40 fibrils could potentially arise from the intramolecular ionic interaction between the side chain of pS8 and H6, which is partially disrupted at high pressure and, because of the electrostriction of solvent molecules by separated charges, could lead to a volume decrease without Aβ monomer release^29^.

Despite only two additional residues at the C terminus, Aβ42 displays aggregation and toxicity properties that strongly exceed those of Aβ40 (ref. 34). In addition, Aβ42 and Aβ40 follow different aggregation pathways^41^ and populate distinct oligomeric and fibrillar states^42^,^43^. Using high-pressure NMR we here showed that aggregates of Aβ42 are kinetically and thermodynamically more stable than those of Aβ40 (Fig. 3b). In particular, the smaller off-rate of Aβ42 assemblies indicates that the lifetime of these potentially toxic Aβ aggregates is significantly prolonged and might be an important factor that contributes to the toxic role of Aβ42 in AD. Further studies are required to explain the structural basis of the enhanced stability of Aβ42 assemblies.

A key step in the prion-like spreading of neurodegenerative pathology is the seeding of soluble protein by previously formed and transmitted protein aggregates^44^. As shown for the yeast prion protein, seed generation is controlled by the rate at which amyloid fibrils fragment, which is in turn governed by their stability^15^. Because aggregation seeds have to be transmitted to the next cell and different brain areas, the efficiency of spreading relies on the stability of aggregation seeds. In case of Aβ, it is known that multiple states of Aβ assemblies are capable of inducing amyloid aggregation *in vivo*, suggesting that small soluble Aβ seeds are potent mediators of the spreading of extracellular β-amyloidosis in AD^45^. Our data demonstrate that phosphorylation enhances the stability of Aβ assemblies (Figs 1, 3 and 6). In addition, the dissociation rate of monomeric Aβ from phosphorylated Aβ aggregates is reduced, which prolongs the lifetime of phosphorylated Aβ assemblies. Because pS8Aβ assemblies can cross-seed the aggregation of non-phosphorylated Aβ (ref. 22), the prolonged lifetime of S8-phosphorylated Aβ aggregates might increase spreading of AD pathology.

In summary, our study demonstrates that phosphorylation of Aβ at serine 8 enhances the stability and prolongs the lifetime of Aβ aggregates. Serine 8 is located in the N-terminal region of Aβ and therefore likely to be exposed in amyloid deposits and thus available to the enzymatic machinery of phosphorylation. Importantly, our work indicates that phosphorylation changes the molecular properties of protein aggregates even after they are formed. Phosphorylation and potentially other post-translational modifications can thus modulate aggregate-induced toxicity in the human brain.

## Methods

### Materials

Aβ40 and Aβ42, as well as Aβ40 phosphorylated at serine 8 (pS8Aβ) were obtained from Peptide Specialty Laboratory (Germany) and used without further purification. The Aβ40 variant S8D (S8DAβ) was purchased from EZBiolab (USA). The monoclonal Aβ antibody 82E1 was obtained from IBL Corporation (Japan). Unless stated otherwise, Aβ stock solutions were prepared by dissolving peptide powders in 10 mM NaOH at a concentration of 2 mg ml^−1^ (∼460 μM), then flash freezing the aliquots of Aβ stock solutions in liquid nitrogen and storing them at −80 °C until further use. Stock solutions of Aβ42 were prepared by a two-step solubilization method^46^.

### PAGE and western blotting

npAβ and pS8Aβ (50 μM, pH 7.4 buffered with 20 mM sodium phosphate containing 50 mM sodium chloride) were incubated at 37 °C with gentle stirring. Aliquots of samples collected at different time points were centrifuged at 100,000*g* for 30 min to separate the soluble Aβ species in the supernatant from insoluble Aβ assemblies in the pellet. The supernatant and pellets were subjected to native-PAGE or denaturing SDS–PAGE and Aβ was detected by western blotting. The samples were mixed with an equal volume of 4 × SDS sample buffer and then boiled at 100 °C for 5 min. The samples were electrophoresed on 4–12% Bis-Tris gels (Novex, San Diego, CA, USA) and then transferred onto 0.2-μM nitrocellulose membrane (Whatmann GmbH, Germany) at 400 mA for 2 h at 4 °C. After 2 h, membranes were washed in TBS-T and incubated overnight with the primary antibody 82E1 in 0.5 μg ml^−1^ TBS-T solution at 4 °C. The bound antibody was detected using horseradish peroxidase-conjugated anti-mouse IgG (1:50,000; Sigma) using enhanced chemiluminescence (ECL reagent, GE Healthcare) with an ECL imager (BioRad Inc.).

### Electron microscopy

npAβ, pS8Aβ and S8DAβ samples of 0.2 mg ml^−1^ (∼50 μM) concentration in HEPES buffer (25 mM, pH 7.4, 50 mM NaCl) were incubated at 37 °C with gentle stirring. After 48 h of incubation, samples were diluted, deposited onto carbon-coated copper mesh grids and negatively stained with 2% (w/v) uranyl acetate. Excess stain was washed away, and the sample grids were allowed to air dry. Samples were examined using a Philips CM 120 BioTwin transmission electron microscope (Philips Inc., Eindhoven, The Netherlands). The apparent diameter (largest width) of negatively stained Aβ fibrils were measured using the ITEM software (Olympus Soft Imaging Solutions, Münster, Germany) and the size distribution of npAβ and pS8Aβ fibrils was determined after manual measurement of >300 fibrils per peptide variant.

To investigate the effect of high pressure on Aβ fibrils, npAβ and pS8Aβ samples of 0.25 mg ml^−1^ (∼60 μM) concentration in phosphate buffer (50 mM, pH 7.4, 50 mM NaCl) were incubated in aggregation-prone condition (37 °C, with gentle stirring) for 96 h. Before and after application of high hydrostatic pressure (2,000 bar, 18 h) during which the pressure-induced Aβ monomer release was monitored, Aβ samples were taken for the EM examination. The EM images were obtained as described above.

### High-pressure NMR experiments

npAβ40, pS8Aβ40, S8DAβ40 and Aβ42 samples of 0.25 mg ml^−1^ (∼60 μM) concentration in phosphate buffer (50 mM, pH 7.4, 50 mM NaCl, 100 μM DSS, 10% D_2_O) were incubated in aggregation-prone condition (37 °C, with gentle stirring). After the 96 h of incubation, the aggregated Aβ samples were transferred without further fractionation to high-pressure NMR tubes and subjected to hydrostatic pressures up to 2,000 bar (Daedalus Innovations LLC, PA) and low temperature (273 K). NMR experiments were performed on a 700 MHz Bruker spectrometer (Germany). At each pressure, Aβ samples were first incubated for 20 min, then three consecutive 1D proton experiments each taking 10 min were recorded, before raising the pressure to the next higher level. The amount of Aβ monomer released as a function of the applied pressure was estimated through proton signal intensities in the methyl region, normalized by the intensity of an internal DSS signal. For the kinetics of pressure-induced Aβ monomer release from aggregates, the aggregated Aβ samples were directly subjected to the highest pressure, that is, 2,000 bar, and 1D proton NMR spectra were measured for 18 h at 30-min intervals. The time-dependent relative intensity data (*I*) were fitted to mono- and bi-exponential equations:


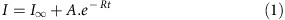



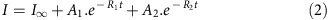


Assuming a simple model for the conversion between NMR-invisible Aβ assemblies (*A*) and monomers (*M*):


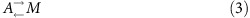


in which both the forward dissociation and backward association reactions obey first-order kinetics governed by the rate constants *k*_off_ and *k*_on_. The fitting parameters derived from the monoexponential equation (equation (1)) were interpreted as:


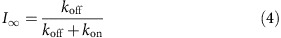



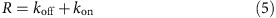


and apparent values of *k*_off_ and *k*_on_ were derived accordingly. The assumption of (pseudo) first-order kinetics for the reverse reaction in equation (3) is reasonably justified noting that the number of growing ends in Aβ aggregates does not change during dissociation–association reaction. The fitting parameters, which were derived from the bi-exponential model (equation (2)), were interpreted according to the following model:


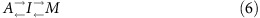


which involves reversible conversion of Aβ assemblies to an intermediate state (I) before the reversible monomer dissociation–association reaction occurs. An in-house Mathematica 8.0 (Wolfram Research, Inc.) script was used for data fitting.

To obtain the change in volume on Aβ monomer release from aggregates, the npAβ40 and pS8Aβ40 samples (0.25 mg ml^−1^ in 50 mM phosphate, pH 7.4, 50 mM NaCl, 100 μM DSS, 10% D_2_O) were incubated in aggregation-prone conditions (37 °C, with gentle stirring) for 96 h. Subsequently, they were transferred to high-pressure NMR tubes and incubated for 48 h at different pressure levels (500, 1,000, 1,500 and 2,000 bar) and 273 K. The 1D NMR proton spectra were recorded before aggregation (day 0), after aggregation (day 4) and after 48-h application of high pressure to Aβ aggregates. Aβ monomer levels were quantified as described above, the equilibrium constants between Aβ aggregates and monomers 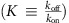 were calculated according to equation (3) and then converted to free-energy change on Aβ monomer release from aggregates (Δ*G*):


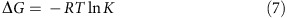


in which *R* is the ideal gas constant and *T* is (absolute) temperature. Assuming that the pressure dependence of free-energy change is governed by the following linear relation,


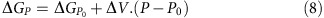


the (apparent) volume change on Aβ monomer release from aggregates (Δ*V*) was estimated from the slope of the fitted Δ*G* versus pressure line.

### MD simulation

MD simulations were performed using the GROMACS simulation package, version 4.6.5 (ref. 47). The Amber99sb force field parameters were used as implemented in GROMACS^48^. The initial protein model was based on a solid-state NMR structure of Aβ40 fibrils (PDB code: 2M4J (ref. 33)). This protein database (PDB) structure contains three layers of a threefold symmetric repeat unit each constituted by three Aβ molecules. Taking the lowest-energy model of this PDB entry, three models were built for the unmodified, S8-phosphorylated and S8D-Aβ40 fibrils. To reduce the contribution of end effects in the final results, each model was elongated by adding new layers in the direction of the fibrillar axis. The final models were 21-layered structures of Aβ40 fibrils, composed of a total of 63 polypeptide chains. Each fibril model was solvated in a cubic box of dimension 11 × 11 × 11 nm^3^ with TIP4P-Ew water molecules and neutralized by adding monovalent ions. To remove atomic clashes, each solvated and neutralized model was then subjected to energy minimization using the steepest descent algorithm until the maximum force on atoms was reduced to values smaller than 1,000 kJ mol^−1^ nm^−1^. After energy minimization, short-length position-restrained NVT and position-restrained NPT simulations (1 ns) at 300 K were applied to set atomic velocities and adjust densities. After equilibration, the production MD simulations were performed for each model at constant temperature (300 K) and pressure (1 bar) over a 75-ns period. The LINCS algorithm was used to constrain the bonds. Lennard–Jones and short-range electrostatic interaction cutoff radii of 1 nm were used. Coulomb interactions at longer distances were calculated by the Particle Mesh Ewald algorithm. The protein fibril and water molecules plus ions were coupled separately to a thermal bath using the velocity rescale algorithm (modified Berendsen thermostat) with *τ*_*T*_=0.1 ps at 300 K (refs 49, 50). The isotropic pressure coupling was used to keep the pressure at desired values by applying the Berendsen algorithm with *τ*_*P*_=2.0 ps and a compressibility of 4.5 × 10^−5^ bar^−1^ (ref. 50). The neighbour lists were updated every five steps and a time step of 2 fs was chosen for integration. Hydrogen bonds were calculated using the angle and distance cutoff values of 30° and 0.35 nm, respectively.

To evaluate the impact of high pressure on Aβ fibrils, two additional 75-ns MD simulations were performed on unmodified and S8-phosphorylated Aβ40 fibrils using the same set-up as mentioned above except of pressure and temperature which were set to 2,000 bar and 273 K in accordance with the experimental condition of our high-pressure NMR experiments. Packing efficiencies were then calculated as the ratio of the van der Waals volume to the Voronoi volume over the last 25 ns of MD trajectories, using the computational tool of *trjVoronoi* (ref. 51).

Details on ThT binding assays are provided in Supplementary Methods.

## Additional information

**How to cite this article:** Rezaei-Ghaleh, N. *et al*. Phosphorylation modifies the molecular stability of β-amyloid deposits. *Nat. Commun.* 7:11359 doi: 10.1038/ncomms11359 (2016).

## Supplementary Material

Supplementary Information Supplementary Figures 1-7, Supplementary Tables 1-2, Supplementary Methods and Supplementary Reference
