# Supplementary material for: Phosphorylation modifies the molecular stability of β-amyloid deposits
Source: Nat Commun. 2016 Apr 13;7:11359. doi: 10.1038/ncomms11359 (PMC4833870; doi:10.1038/ncomms11359)
Supplement: Supplementary Information — Supplementary Figures 1-7, Supplementary Tables 1-2, Supplementary Methods and Supplementary Reference [file ncomms11359-s1.pdf]

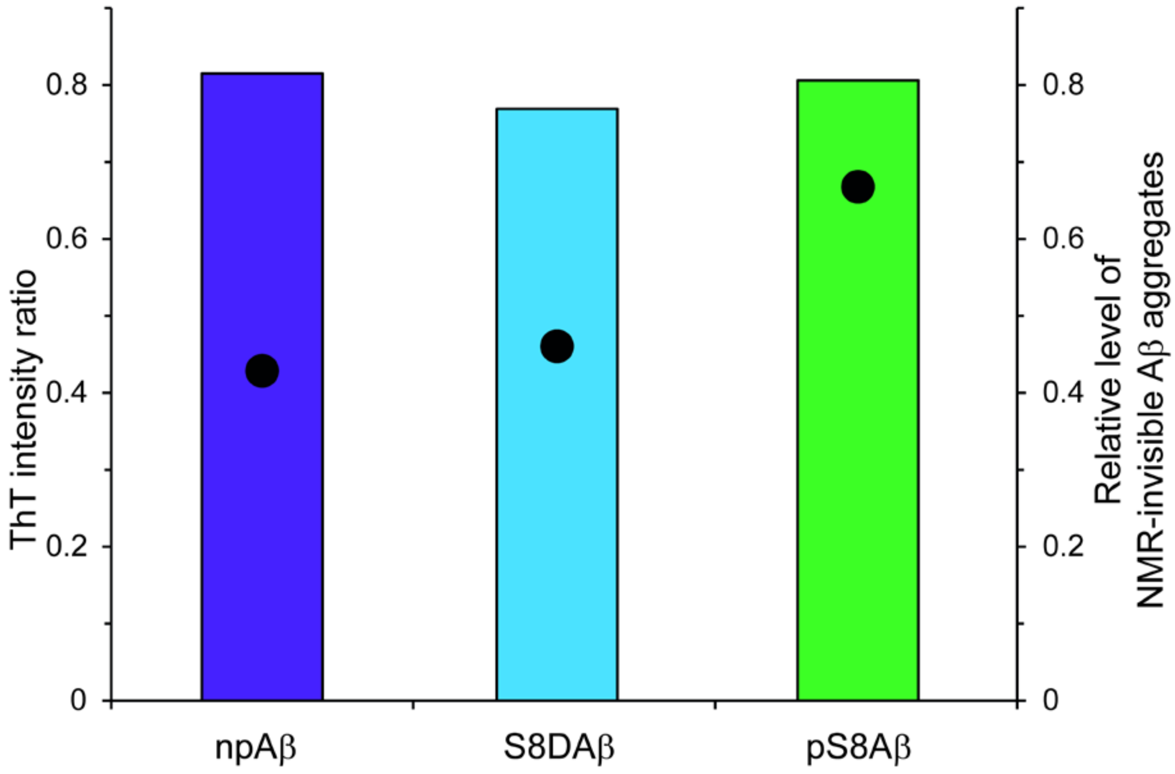

Supplementary Figure 1. Application of high pressure (2000 bar, 18 hours) to aggregated npAβ, pS8Aβ and S8DAβ reduced their ThT fluorescence intensity to ~ 80% of the initial levels before pressure application. The drop in ThT intensity is less prominent than the pressure-induced decrease in the NMR-invisible Aβ aggregates (second axis, represented by solid circles).

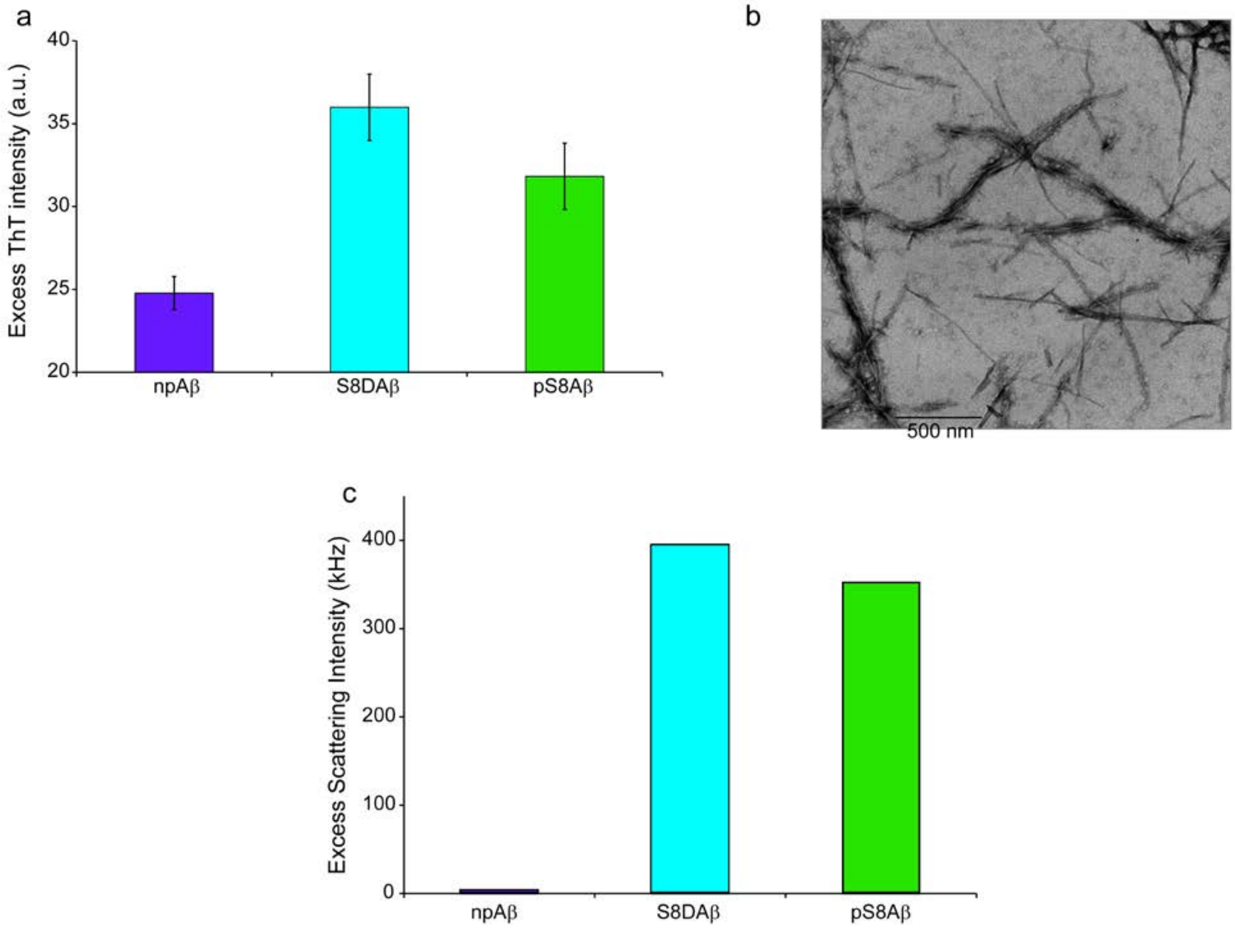

Supplementary Figure 2. Mutation of serine 8 to asparate mimics the higher propensity of S8-phosphorylated Aβ for fibrillar and oligomeric aggregation. **(a)** Thioflavin T (ThT) fluorescence intensity of specified Aβ variants after 48 hours of aggregation. Both S8DAβ and pS8Aβ demonstrate higher propensity to form ThT-reactive aggregates than npAβ, in accord with time-dependent ThT data previously reported in [1]. ThT values are reported after subtraction of the control intensity, and error bars represent the standard deviation of triplicate experiments. **(b)** Electron micrograph of S8DAβ fibrillar aggregates. **(c)** Excess scattering intensity of the supernatant of aggregated Aβ solutions following centrifugation (16,000 g, 20 minutes) of the 48-hours aggregated Aβ samples. The larger values of the scattering intensity of S8DAβ and pS8Aβ samples indicate their higher tendency than npAβ to form soluble assemblies.

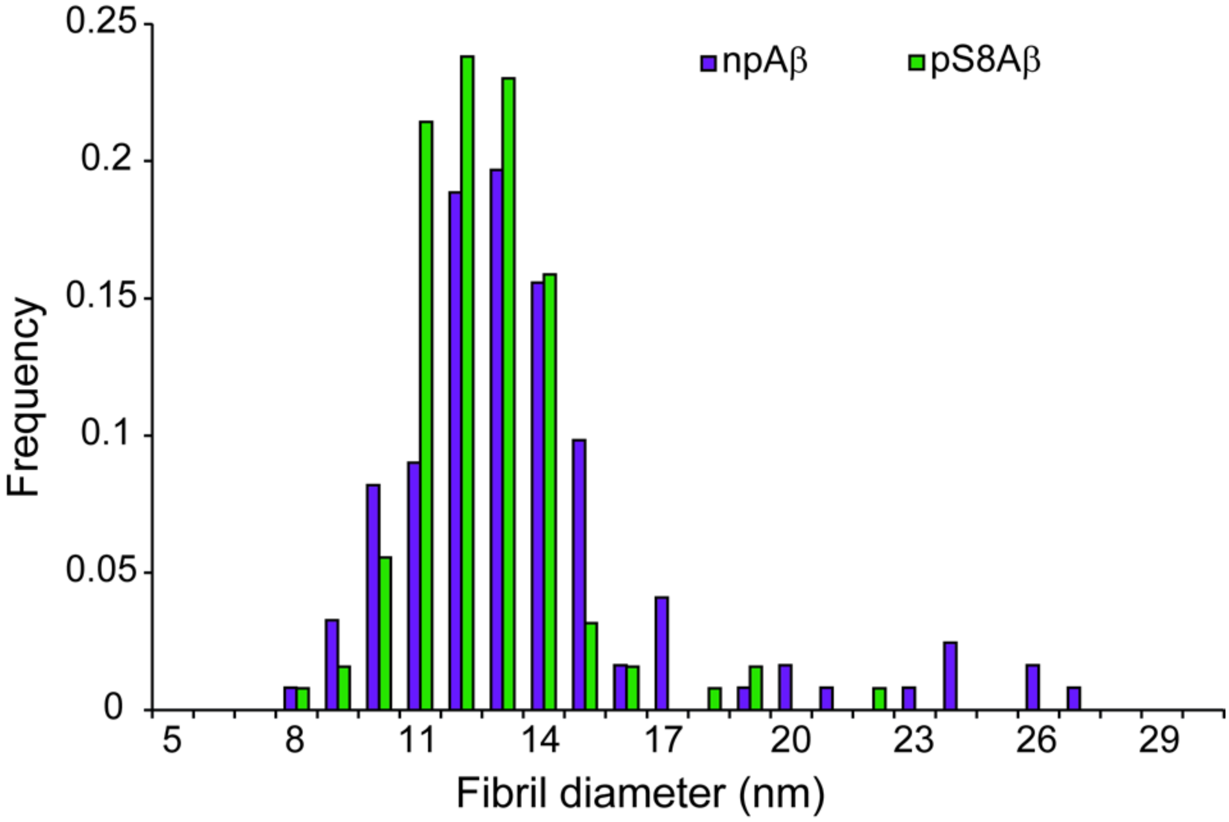

Supplementary Figure 3. Size distribution of npAβ and pS8Aβ fibrils, obtained from measurement of more than 300 fibrils per peptide variant. Phosphorylation of Aβ at serine 8 only slightly changed the diameter of Aβ fibrils.

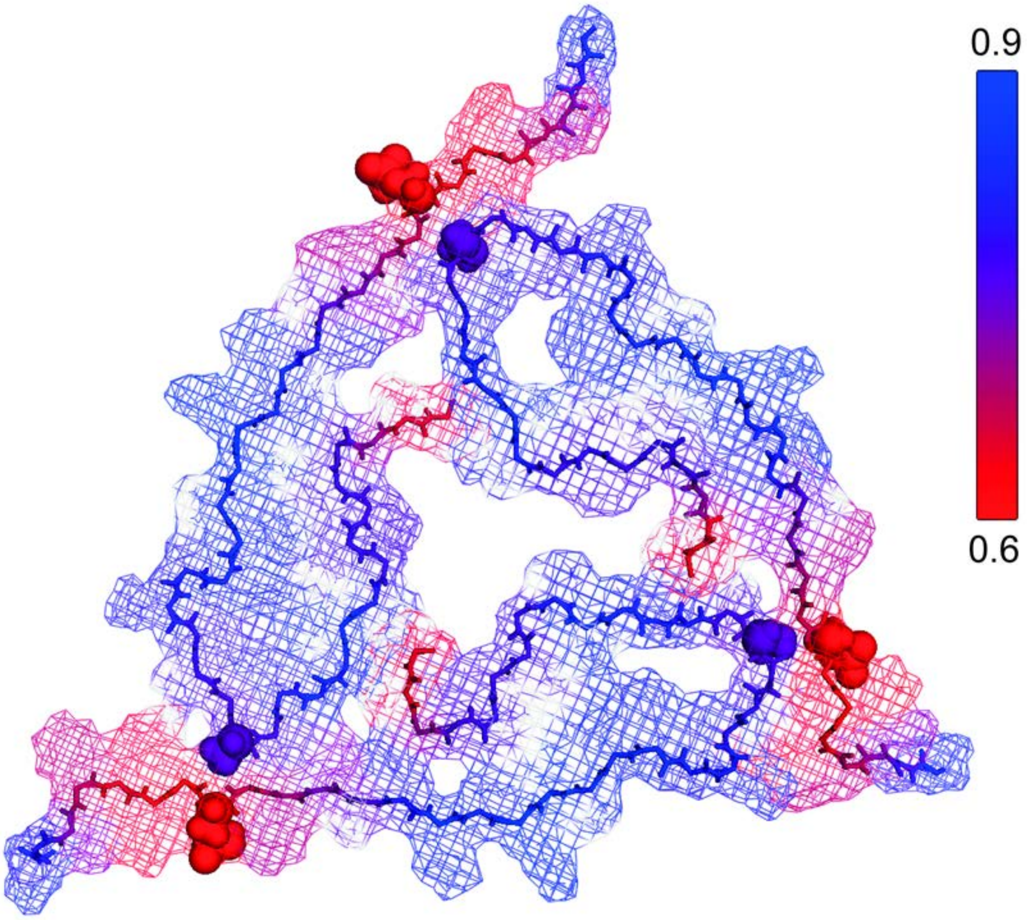

Supplementary Figure 4. The ratio of the backbone RMSF of pS8Aβ to npAβ fibrils is mapped onto the three-dimensional structure of Aβ40 fibrils (average structure of MD trajectory). Regions shown in red (see color scale) are less mobile in Aβ40 fibrils phosphorylated at serine 8. The side chains of pS8 and S26 are displayed by spheres.

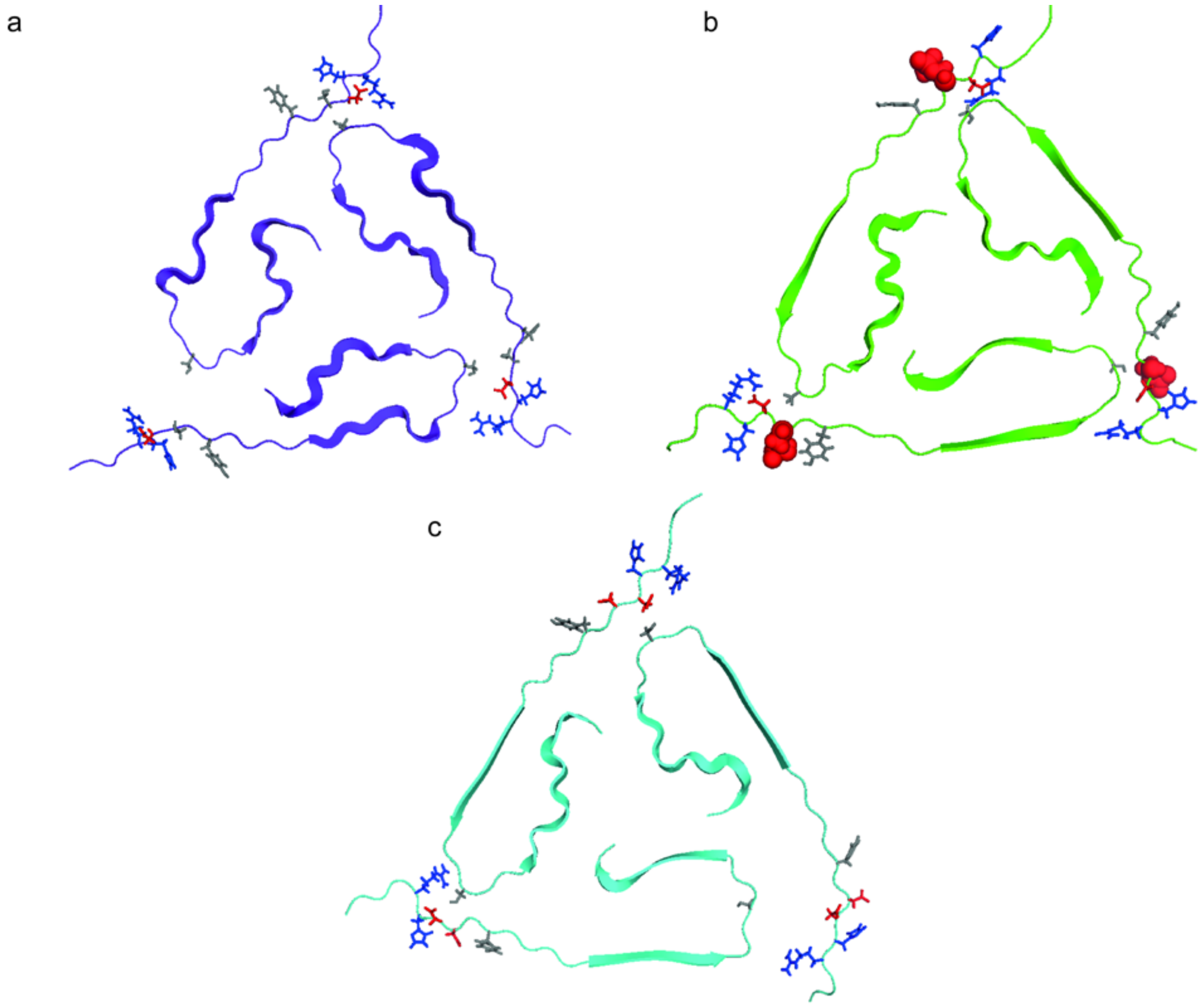

Supplementary Figure 5. MD average structure of npAβ (a), pS8Aβ (b) and S8DAβ (c) fibrils. Residues R5, H6, D7, S8, D8, Y10 and S26 are color-coded on the basis of their side chain charge. The side chains of pS8 (b) are displayed by red spheres.

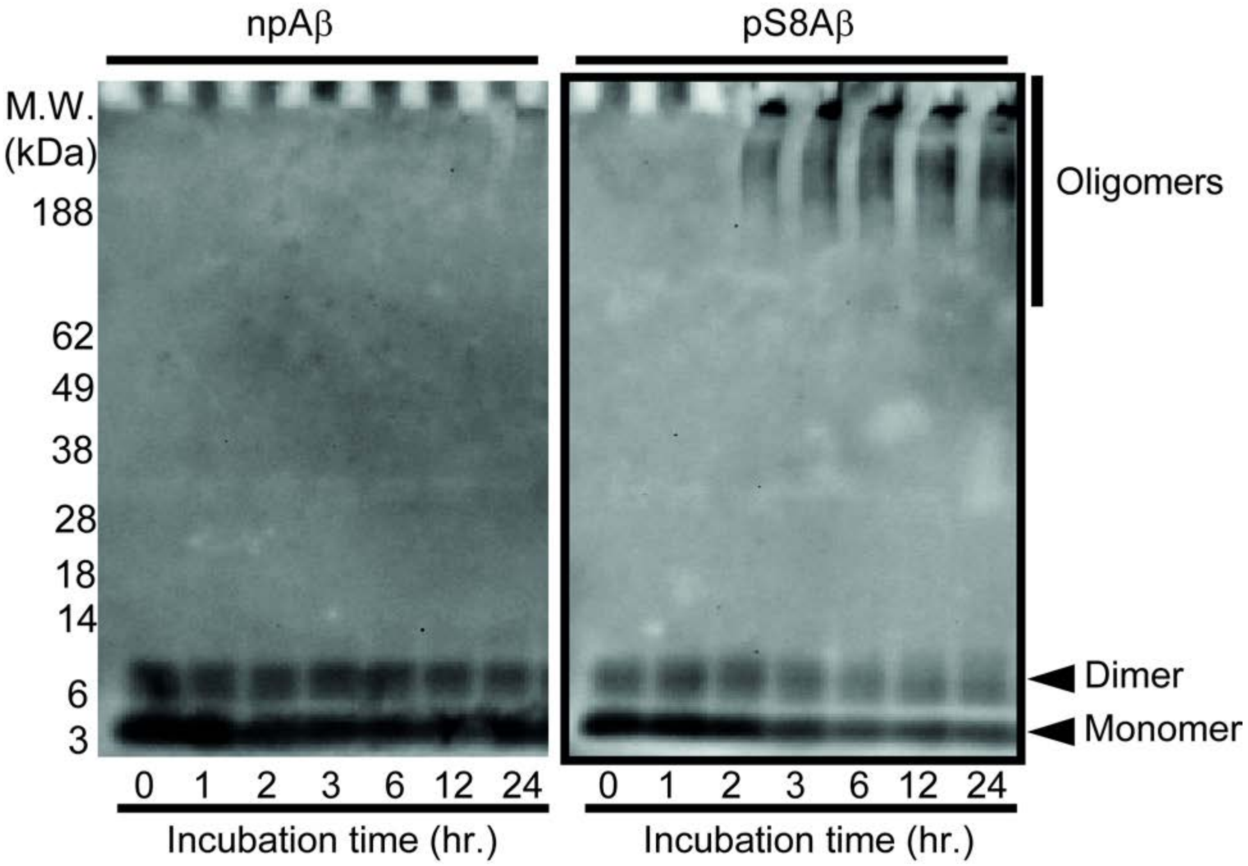

Supplementary Figure 6. Phosphorylation of Aβ at serine 8 enhances the stability of Aβ oligomers. npAβ and pS8Aβ (50 μM in 20 mM sodium phosphate buffer, pH 7.40, containing 50 mM NaCl) were incubated at 37 °C with gentle stirring. Aliquots of samples were collected at different time intervals, the whole aliquots were subjected to denaturing SDS-PAGE and Aβ was detected by Western-blotting. The high-molecular weight oligomers of pS8Aβ showed remarkable resistance against SDS-induced dissociation.

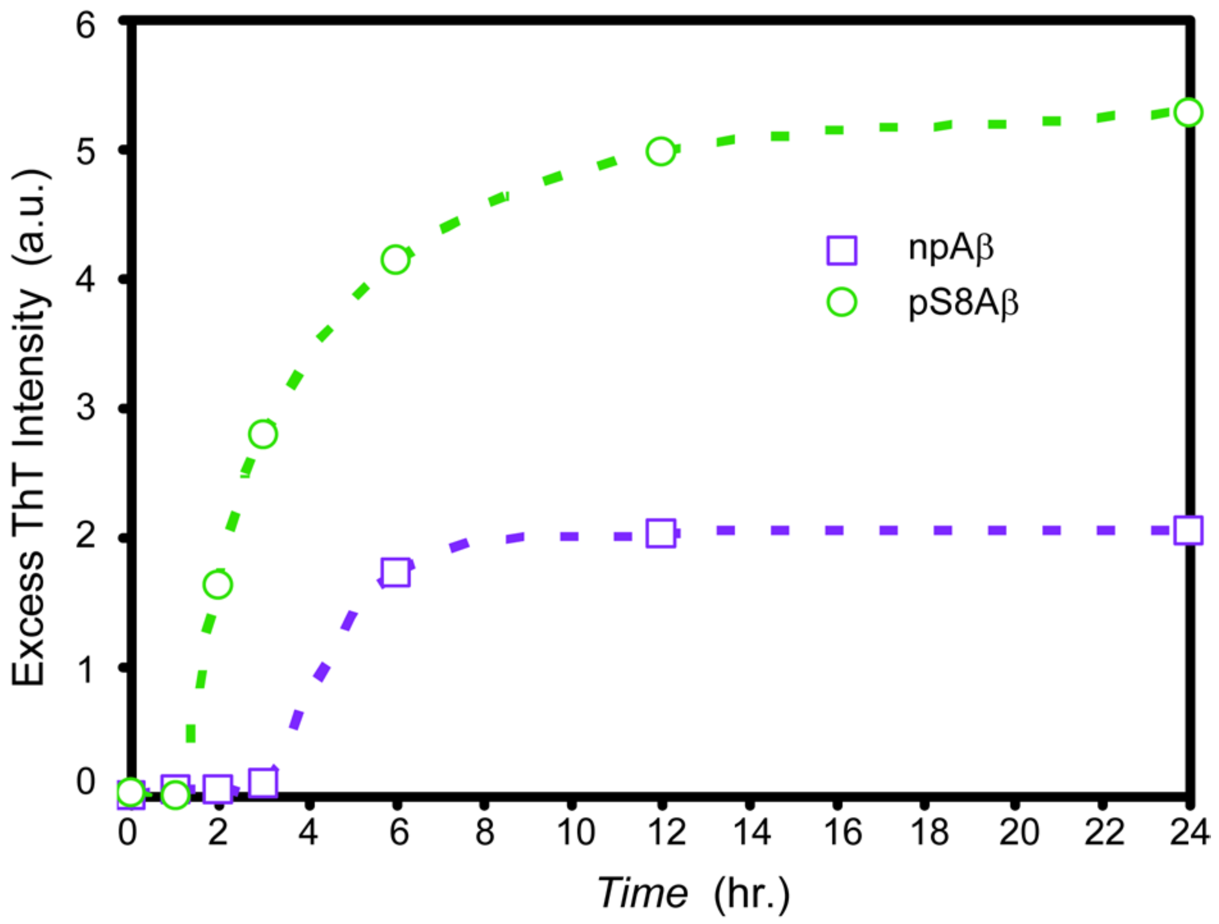

Supplementary Figure 7. Temporal evolution of ThT fluorescence intensity during aggregation of Aβ samples used for denaturing SDS-PAGE experiments (shown in **Fig. 6** and **Supplementary Fig. 6**).

Supplementary Table 1. Fitting parameters obtained from the analysis of Aβ monomer release from aggregates at high hydrostatic pressure (2000 bar).

	**npAβ**	**S8DAβ**	**pS8Aβ**	**Aβ42**
**mono-exponential fit**				
I_{∞}	0.62±0.00*	0.55±0.00	0.43±0.00	0.33±0.00
$-A$	0.35±0.01	0.37±0.01	0.21±0.00	0.20±0.00
R (10^{-4} s^{-1})	0.79±0.04	0.74±0.03	0.73±0.04	0.30±0.02
*SSE***	0.002993	0.0027	0.001137	0.0006878
Adjusted *R-square*	0.9879	0.9906	0.9883	0.9945
**bi-exponential fit**				
I_{∞}	0.66±0.01	0.59±0.01	0.45±0.00	0.37±0.00
$-A_1$	0.21±0.01	0.23±0.01	0.16±0.01	0.21±0.00
R_1 (10^{-4} s^{-1})	0.33±0.04	0.32±0.05	0.44±0.04	0.18±0.01
$-A_2$	0.24±0.01	0.24±0.02	0.12±0.01	0.05±0.00
R_2 (10^{-4} s^{-1})	2.21±0.19	1.91±0.20	2.99±0.50	2.13±0.18
*SSE***	0.0002066	0.0002642	0.0002099	0.0000337
Adjusted *R-square*	0.9991	0.9990	0.9977	0.9997
*F-statistic****	202.3	138.3	68.5	378.5
P-value	<0.0001	<0.0001	<0.0001	<0.0001

*. Errors in the fitting parameters were derived from covariance matrices.

**.*SSE*: Sum of Squares due to error.

***. *F-statistic*, computed between mono- and bi-exponential fits.

Supplementary Table 2. Fitting parameters obtained from the analysis of pressure-induced Aβ monomer release data (2000 bar) according to two physical models.

	**npAβ**	**S8DAβ**	**pS8Aβ**	**Aβ42**
**Model 1***				
k_{off} (10^{-4} s^{-1})	0.49±0.02**	0.41±0.02	0.32±0.01	0.10±0.00
$k_{on,app}$ (10^{-4} s^{-1})	0.30±0.02	0.33±0.02	0.42±0.02	0.20±0.01
*SSE***	0.002993	0.002700	0.001137	0.000688
Adjusted *R-square*	0.9879	0.9906	0.9883	0.9945
**Model 2***				
k_1(10^{-4} s^{-1})	0.30±0.05	0.29±0.06	0.36±0.10	0.15±0.01
k_2 (10^{-4} s^{-1})	0.42±0.07	0.45±0.07	0.86±0.14	0.02±0.01
k_{off} (10^{-4} s^{-1})	1.37±0.08	1.06±0.07	0.72±0.09	0.72±0.06
$k_{on,app}$ (10^{-4} s^{-1})	0.29±0.03	0.28±0.04	0.25±0.06	1.05±0.09
*SSE****	0.000210	0.000266	0.000242	0.000037
Adjusted *R-square*	0.9991	0.9990	0.9973	0.9997
*F-statistic*****	128.16	88.42	36.96	221.84
P-value	<0.0001	<0.0001	<0.0001	<0.0001

*. Please see Fig. 3 for description of models 1 and 2.
**. Errors in the fitting parameters were derived from covariance matrices.
***.*SSE*: Sum of Squares due to error.
****. *F-statistic*, computed between Models 1 and 2.

Supplementary Methods

Thioflavin T (ThT) dye binding assays. 50 μM solutions of npAβ and pS8Aβ in 20 mM sodium phosphate buffer (pH 7.4, containing 50 mM NaCl) were incubated at 37 °C with gentle stirring. The ThT binding assay was performed by mixing 10 μL aliquots with 400 μL of 20 μM ThT solutions followed by fluorescence measurements using a Cary Eclipse spectrophotometer (Varian, Australia). ThT fluorescence emission intensities were measured at 482 nm following excitation at 446 nm. All measurements were performed in triplicates.

Supplementary Reference

1. Kumar S, *et al.* Extracellular phosphorylation of the amyloid beta-peptide promotes formation of toxic aggregates during the pathogenesis of Alzheimer's disease. *EMBO J.* **30**, 2255-2265 (2011).
